# Retraction Note: Individual level and community level factors affecting exclusive breast feeding among infants under-six months in Ethiopia using multilevel analysis

**DOI:** 10.1186/s13052-024-01699-6

**Published:** 2024-07-23

**Authors:** Shambel Aychew Tsegaw, Yeshimebet Ali Dawed, Erkihun Tadesse Amsalu

**Affiliations:** 1 Department of Public health, Dessie Health science college, Dessie, Ethiopia; 2 Department of Nutrition, school of Public Health, College of Medicine and Health Sciences, Wollo University, Dessie, Ethiopia; 3 Department of Epidemiology and Biostatistics, School of Public Health, College of Medicine and Health Sciences, Wollo University, Dessie, Ethiopia

**Retraction Note: Italian Journal of Pediatrics (2021) 47:106**

10.1186/s13052-021-01062-z

The Editor-in-Chief has retracted this article because this article is a duplicate publication of another article published in PLoS ONE [1] by the same authors.

Author, Yeshimebet Ali Dawed agrees to this retraction. Authors, Shambel Aychew Tsegaw and Erkihun Tadesse Amsalu have not responded to any correspondence from the editor/publisher about this retraction.
